# Development of a Novel Shock Wave Catheter Ablation System -The First Feasibility Study in Pigs-

**DOI:** 10.1371/journal.pone.0116017

**Published:** 2015-01-29

**Authors:** Yuhi Hasebe, Hiroaki Yamamoto, Koji Fukuda, Kensuke Nishimiya, Kenichiro Hanawa, Tomohiko Shindo, Masateru Kondo, Makoto Nakano, Yuji Wakayama, Kazuyoshi Takayama, Hiroaki Shimokawa

**Affiliations:** 1 Department of Cardiovascular Medicine, Tohoku University Graduate School of Medicine, Sendai, Japan; 2 Department of Advanced Cardiovascular Medicine, Tohoku University Graduate School of Medicine, Sendai, Japan; University of Minnesota, UNITED STATES

## Abstract

**Introduction:**

Radio-frequency catheter ablation (RFCA) using Joule heat has two fundamental weaknesses: the limited depth of treatment and the risk of thrombus formation. In contrast, focused shock wave (SW) therapy could damage tissues at arbitrary depths without heat generation. Thus, we aimed to develop a SW catheter ablation (SWCA) system that could compensate for the weaknesses of RFCA therapy.

**Methods and Results:**

We developed a SWCA system where the SW generated by a Q-switched Holmium: yttrium aluminum garnet (YAG) laser beam was reflected by a reflector attached to 14-Fr catheter tip and then was converged onto the focus. We examined the feasibility of our system on pigs in vivo. When applied using the epicardial approach, the SWCA caused persistent spheroidal lesions with mild superficial injury than the RFCA. The lesions were created to a depth based on the focal length (2.0 mm) [2.36 ± 0.45 (SD) mm immediately after procedure, n = 16]. When applied to the atrioventricular (AV) node using the endocardial approach, the SWCA caused junctional escape rhythms in 2 pigs and AV block in 12 pigs (complete AV block in 9) in acute phase (n = 14). Nine of the 14 pigs survived with pacemakers for the long-term study, and the AV block persisted for 12.6 ± 3.9 (SD) days in all surviving pigs. Histological examination showed AV nodal cell body atrophy in the acute phase and fibrotic lesions in the chronic phase. Importantly, no acute or chronic fatal complications were noted.

**Conclusions:**

Our novel SWCA system could be a promising modality as a non-thermal ablation method to compensate for the weaknesses of RFCA therapy. However, further research and development will be necessary as the current prototype still exhibited the presence of micro-thrombus formation in the animal studies.

## Introduction

Supraventricular tachyarrhythmias, including atrial fibrillation, are associated with decreased quality of life, a risk of thromboembolism, and heart failure [1], [2]. On the other hand, ventricular arrhythmias (VAs) can cause sudden cardiac death (SCD), particularly in patients with structural heart diseases [3]. Antiarrhythmic drugs can be effective treatment for many types of tachyarrhythmias, but can occasionally be associated with resistance, unfavorable side effect profiles, and pro-arrhythmic risks. Implantable cardioverter defibrillators (ICDs) have also been an effective modality to reduce SCD caused by malignant Vas; however, frequent VAs and ICD shocks have also been associated with increased mortality [4].

In the past 20 years, radio-frequency catheter ablation (RFCA), which selectively necrotizes arrhythmogenic tissues, has become an established and curative therapeutic modality for drug resistant tachy-arrhythmias. With new technological developments and the addition of ablation strategies, including electroanatomical mapping systems and substrate-based ablation procedures, life-threatening hemodynamically unstable ventricular tachycardias (VTs) have become candidates for the RFCA [5], [6]. However, the RFCA has two fundamental weaknesses, including the limited depth of treatment and the risk of thrombus formation, as it creates coagulation lesions using Joule heat [7–9]. The limited depth due to the thermal conductivity of the tissue is one of the reasons that the RFCA of VTs may encounter difficulties, particularly in patients with structural heart diseases. Indeed, it has been reported that an epicardial approach would be required to eliminate VTs in 23% of patients with ischemic cardiomyopathy [10] and in 35% of those with non-ischemic cardiomyopathy [11]. Furthermore, VTs originating from the ventricular septum (e.g., hypertrophic cardiomyopathy) are often refractory to the RFCA even with the combination of the endo- and epicardial approach due to the deep intramural arrhythmic focus [12], [13]. On the other hand, the risk of thrombus formation during the RFCA is caused by the endothelial disruption, coagulation necrosis, and the heating of circulating blood elements, resulting in fatal or non-fatal thromboembolic complications such as a cerebral stroke [9]. The risk of symptomatic stroke associated with the RFCA was 0.6% and increased to 1.8%–2% for procedures on the left side of heart [9]. In addition, a non-negligible risk of silent cerebral ischemia detected by cerebral magnetic resonance imaging (MRI) has been recognized [14].

Since its introduction in the 1980s, extracorporeal shock wave lithotripsy (ESWL) has been a well-established treatment option for urinary stones [15], [16]. Focused SW has also been used for heart therapies. We have previously demonstrated that low-energy extracorporeal SW therapy can be an effective and non-invasive angiogenic therapy for ischemic heart disease without causing myocardial tissue injury [17], [18]. On the other hand, focused SW could cause tissue damage at an arbitrary depth without heat generation because damage to the renal tissue near the SW focus site in ESWL has also been reported [19]. Although the biological effect of high-energy SW application directly to the myocardium remains to be clarified, if the focused high-energy SW caused adequate persistent myocardial tissue damage to ablate the arrhythmogenic substrate, it may be an ideal energy source for a non-thermal catheter ablation system that could reduce thrombus formation. In addition, if the SW could be focused onto areas that the RFCA could not reach, it would improve the treatment outcomes of the RFCA for refractory VTs.

In the present study, our goal was to develop a novel SW catheter ablation (SWCA) system that could generate high-energy focused SW equivalent to ESWL and to examine its feasibility and safety in pigs in vivo.

## Materials and Methods

### Examination of Characteristics of SW Generated by the SWCA System

We examined the basic characteristics of SW generated by the SWCA system in degassed saline *in vitro*. The shadowgraph of SW was taken by a high-speed camera (SIM02, Specialized Imaging Ltd., Tring, UK). The pressure distribution of the SW was measured using a polyvinylidene fluoride (PVDF) needle hydrophone with a 0.5 mm sensitive diameter and a 35 ns rise time (Dr. Müller Instruments Inc., Oberursel, Germany). The signals were stored in the digital transient memory (TDS3014B, Tektronix Inc., Oregon, USA) at a sampling rate of 100 MHz.

We calculated the acoustic pulse integral intensity (PII) [20] at the focal site according to the pressure waveform and it was defined as follows:

$$\text{PII}=\frac{1}{\rho c}\overset{t_{2}}{\underset{t_{1}}{\int }}p^{2}\left(\text{t}\right)\text{dt}\;\left(\text{J}/{\text{m}}^{2}\right)$$

The variable t_1_ was the time just before when the pulse began, t_2_ represented just after when the pulse ended, ρc represented the characteristic acoustic impedance of water (1.5 × 10^6^ N・s/m3), and p(t) was the instantaneous acoustic pressure.

### Feasibility Studies in Pigs in Vivo

We performed three protocols in pigs *in vivo*. First, we examined whether the focused SW could cause myocardial tissue damages leading to permanent myocardial lesions. We applied the focused SW to the ventricular myocardium under direct vision using the epicardial approach and analyzed the treated sites both macroscopically and histologically. Second, we examined whether an endocardial SW application could also cause ventricular myocardial lesions as with the epicardial approach. We applied the focused SW to the right ventricular (RV) myocardium with the percutaneous transcatheter endocardial approach and analyzed the treated sites. Third, we examined whether the myocardial lesions induced by SW could be effective for causing electrophysiological property changes, ultimately by ablating arrhythmogenic tissues. We applied the focused SW to the atrioventricular (AV) node using the endocardial approach via the percutaneous transcatheter technique and analyzed the electrophysiological properties before and after the procedure. All animals received humane care in compliance with the standard guidelines. The present animal study was approved by the committee on Ethics in Animal Experiments of Tohoku University (Nos. 2013MdA-473, 480 and 482).

### Epicardial Ablation Study

We used 27 male domestic pigs (36.6 ± 3.9 kg) in the first protocol (S1 Table). The animals were anesthetized with medetomidine (0.05 mg/kg, IM) and midazolam (0.4 mg/kg, IM). After 10 min, they were intubated after the induction of anesthesia by 5% sevoflurane via inhalation, and were mechanically ventilated with room air and supplemental oxygen. General anesthesia was maintained with 2.5–4.0% sevoflurane. They were continuously monitored with surface electrocardiograms (ECGs), percutaneous oxygen saturation measurements and direct measurements of arterial pressure.

**Fig 1:**
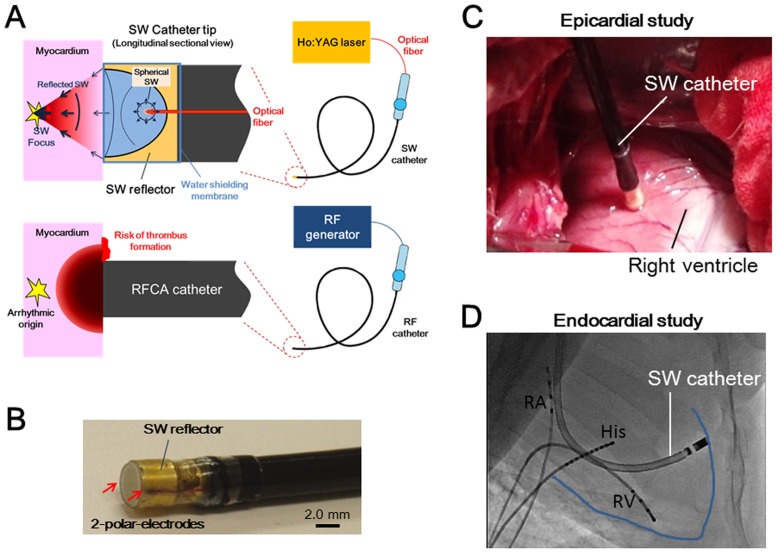
Shock Wave Catheter Ablation (SWCA) System and Catheter Position in Animal Study. The spherical SW was generated in a water-filled semi-elliptical reflector attached to the catheter tip by irradiation of Q-switched Holmium (Ho):yttrium aluminum garnet (YAG) laser beam through quartz optical fiber. The SW was then reflected by the reflector and was converged onto the outer focus. The RF-induced lesion started from the contact area and decreased proportionally with the distance, resulting in a risk of thrombus formation and the inability to reach deep arrhythmic origins (A). The SW catheter was 14-Fr size in diameter and equipped with two polar electrodes at the tip (B). The SW catheter was located vertically to the ventricular myocardium under direct vision in the epicardial ablation study (C). The SW catheter was located as vertically as possible to the right ventricular wall under fluoroscopic vision in the endocardial ablation study (D).

The animals underwent a standard medial sternotomy, and the pericardium was incised in each animal. We applied the focused SW to the right or left ventricular myocardium with the epicardial approach under direct vision. To minimize the mechanical effects of contact with the catheter tip, the SW catheter was gently attached and remained vertical to the ventricular myocardium where coronary vessels or thick epicardial fat tissue did not exist (Fig. 1C). Immediately after the SW application, we marked the near sites by injecting black ink into the epicardium with a 27 gauge needle. We also left plenty of space along each site to prevent intersections between lesions. We used a SW catheter with a 2.0-mm focal length in this study.

First, we applied the focused SW in four different free fields with energy outputs that estimated 0 (sham operation), 20–25, 30–35 and 40–45 MPa to determine the pressure threshold of the myocardial tissue injury [n = 11; total 64 sites; 16 sites at each energy level; 6 in the RV and 10 in the left ventricle (LV)]. The focused SW was applied at a 1 Hz pulse repetition frequency (PRF) against one point for 180 s. Second, we applied SW exceeding 40 MPa to the epicardial ventricular myocardium for three different time durations (30, 60, and 120 s) via 1 Hz to determine the optimal duration of SW application to produce myocardial injury (n = 3; total 48 sites; 16 sites at each durations; 6 in the RV and 10 in the LV). The pigs were euthanized 1 h after the last SW application for the gross and histological examinations for these two protocols during the acute study. We defined a confirmed lesion as the presence of histopathological changes, including myocardial tissue disruption, interstitial hemorrhage, and contraction band necrosis. Third, we performed a survival study to examine whether the SWCA caused irreversible myocardial injury and caused permanent lesions (n = 13). The focused SW was applied to the ventricular myocardium using overpressure that sufficiently exceeded the threshold of myocardial injury at a 1 Hz PRF against one point for 180 s. To examine the time course of the myocardial cell injury by SW application, the surviving animals were subsequently euthanized on Day 1 (n = 3; 16 sites), Day 2 (n = 4; 13 sites), and Day 7 (n = 6; 13 sites) after the procedure. We defined permanent lesions as the presence of fibrosis on the histopathological examination.

For comparison with our SWCA system, we also performed the RF ablation protocols in the same pigs as the SW ablation. We used an open-irrigated-3.5-mm-tip catheter (Thermocool, Biosense-Webster, Inc., Diamond Bar, CA, USA); the RF energy was delivered in a temperature-controlled manner at 43°C and a maximum power output of 30 W for up to 30 s with an irrigation flow of 20 mL/min and limiting impedance decrease of 10 Ω. The animals were euthanized 1 h after the last RF application (n = 4; 16 sites), and the remaining animals were subsequently euthanized on Day 1 (n = 1; 3 sites), Day 2 (n = 1; 2 sites), and Day 7 (n = 1; 3 sites) after the procedure.

Prior to the SW or RF application, bolus amiodarone (150 mg, IV) and lidocaine (25 mg, IV) were administered, and additional bolus lidocaine (25 mg, IV) was injected if VA were observed.

After the *in vivo* experiment, the heart was extracted and subsequently fixed in 10% formalin after the gross examination. The fixed heart was sectioned, and a vertical-section slide was made for each application site.

### Tissue Temperature Measurement Study

We also examined whether the SWCA system could be a non-thermal ablation modality. We applied the focused SW to either the thigh muscle or the epicardial ventricular myocardium and measured the tissue temperature just below the SW reflector in the same pig we utilized for the epicardial ablation study.

An incision was made in the thigh, exposing the muscle. The focused SW was applied to this muscle under direct vision, and the surface temperature just below the catheter tip was continuously measured for 180 s (n = 3). This focused SW was applied with an overpressure that sufficiently exceeded the threshold for myocardial tissue injury at 1 Hz PRF against one point. Furthermore, we performed the same protocol for the epicardial ventricular myocardium (n = 3).

### Endocardial Ventricular Ablation Study

We used other male domestic pigs for the endocardial ventricular ablation study (n = 3, S1 Table). They were anesthetized and monitored as described above. To evaluate the lesions generation by SWCA in the ventricular myocardium with the endocardial approach, we applied the focused SW to the RV free wall (n = 3; 6 sites). The SW catheter was inserted through a percutaneous right femoral vein approach or right jugular vein approach. We situated the catheter as vertically as possible on the RV free wall at the outflow tract or the inferior wall target site (Fig. 1D). The SW catheter used in this study had a focus point of 2.0 mm. The focused SW was applied to one target site at 1 Hz PRF for 180 s. For comparison with the SW lesion, we also performed the RFCA protocol in the same pigs as the SW ablation (n = 3; 6 sites), using a 4-mm-tip catheter (Navister, Biosense-Webster, Inc., Diamond Bar, CA, USA); the RF energy was delivered in a temperature-controlled manner at 50°C and maximum power output of 30 W for up to 60 s with a limiting impedance decrease of 10 Ω. Systemic heparinization by bolus administration (5000 U, IV) followed by continuous infusion (2000 U/h) and additional boluses were performed to maintain an activated clotting time of >300 s during the procedures. The animals were euthanized 1 h after the procedures.

### Endocardial AV Node Ablation Study

We used another 26 male domestic pigs (37.3 ± 7.9 kg) for the endocardial AV node ablation study (S1 Table). They were anesthetized and monitored as described above. Tetra-polar electrodes were placed on the right atrium and right ventricle, and an octa-polar electrode was placed at the bundle of His position through the femoral vein approach. We performed an electrophysiological study (EPS) to analyze the function of the AV conduction system at baseline, including the atrial-His (AH) interval, the His-ventricle (HV) interval, the Wenckebach rate, and the effective refractory period (ERP). The intracardiac ECG was recorded on a polygraph recording system (RMC-3000, Nihon-Kohden, Tokyo, Japan). Then, in the SWCA protocol (n = 14; 5 in the acute study, 9 in the survival study), the SW catheter was inserted through the percutaneous right jugular vein approach and was placed at the target position where His potential could be recorded as close to the atrial side as possible. For fine catheter positioning, we diagnosed the intracardiac potential using a pair of fine-wire electrodes for the electromyography (UNW-2000, Unique Medical Co., Ltd., Tokyo, Japan) that were installed on the lateral side of the SW generator in conjunction with the X-ray fluoroscope. We used a manually curved sheath to direct the reflector of the SW catheter vertically to the target site. We also used a newly developed 3-mm reflector for this endocardial AV node ablation study as the deeper focal length was better for targeting the AV node. The focused SW was applied to one target site at 1 Hz PRF for up to 180 s, and the total SW number was limited to 2,000 shots. The sham operation study was performed in a similar manner (n = 6; 3 in the acute study, 3 in the survival study). The SW catheter was placed at one target site for 180 s without the SW application, and this procedure was repeated five times (for total of 900 s) while slightly changing positions. We also performed the RFCA protocol as a control study (n = 6; 3 in the acute study, 3 in the survival study), using a 4-mm-tip catheter (Navister), and the RF energy was delivered as described above. Systemic heparinization was also performed as described above.

We again performed EPS 15 min after the last application or the sham procedure. The animals were euthanized 1 h after the procedure in the acute study (n = 11; 3 animals in sham operation, 3 in RFCA and 5 in SWCA) or kept alive after pacemaker implantation (Sensia, Medtronic Inc., MN, USA; at VVI 80 beats/min) in the survival study (n = 15; 3 animals in sham operation, 3 in RFCA and 9 animals in SWCA). In the survival study, the animals were continuously monitored by Holter ECG (QR2100, Fukuda M-E Kogyo, Tokyo, Japan) for first 3 days. During Days 7–18, they were again anesthetized, underwent echocardiography and EPS, and were then euthanized.

After euthanasia, gross examination and fixation of the heart was performed as described above, and the specimens were sectioned to include the myocardial lesions at the RV or AV node in a vertical-section slide.

### Histopathological Examination

The tissue specimens were stained with hematoxylin—eosin for all studies, Elastica-Masson stain for the acute study (for clear visualization of the subendothelial layer), and Masson’s trichrome stain for the long-term study (for clear visualization of fibrotic lesions). All histopathological slides were examined by light microscopy (Olympus BX51, Olympus America Inc., NY, USA) and evaluated for myocardial tissue injury, hemorrhage, necrosis, inflammation, thrombus, and fibrosis. In the epicardial study, the superficial tissue damages compared between the SW lesions and the RF lesions by the grading score were determined according to the histological changes of epicardium as follows: intact (no changes, score 0), mild (wavy change with preservation of thickness, score 1), moderate (thinning or partial detachment of epicardium, score 2), and severe (disruption or loss of epicardium, score3). The lesion depth was measured between the myocardial surface and the point where the lesion extended further into the myocardium. The lesion width was measured at the myocardial surface, where it was mostly maximal in the spheroidal lesion. The lesion areas were estimated by plotting the outline of the lesions with image processing software (Axio Vision, Carl Zeiss Inc., Oberkochen, Germany). In the endocardial ablation study, the endothelial tissue damages were compared between the SWCA and RFCA groups by a grading score based on the histological changes of the endothelial cells. The grading score was determined according to the histological changes of endothelial cells as follows: intact (no changes, score 0), mild (wavy change of endothelium, score 1), moderate (crack or partial detachment of endothelium, score 2), severe (loss of endothelium with disruption of subendothelial tissue, score 3). In the AV node ablation study, the four sequential slides that included the AV node and had been sliced at intervals of 1 mm were analyzed for each animal in the acute study (total 20 slides from 5 animals in SWCA; 12 slides from 3 animals in RFCA).

### Statistical Analysis

All continuous data were expressed as mean ± standard deviation (SD). The Mann—Whitney’s U test was used to compare the histological grading score between the SW and RF lesions. One-way analysis of variance (ANOVA) was used to compare data for statistically significant differences, followed by the Tukey’s honestly significant difference (HSD) test to clarify any interactions among the lesion depth, width, and area through the epicardial SW application at each time course. The Student’s t-test was used to compare the lesion depth, width and area between the two groups. A probability of P < 0.05 was considered to be statistically significant.

## Results

### Development of the SWCA System

We developed a novel SWCA system. The schematic of SW generation and convergence onto the focus was shown in Fig. 1A. The system consisted of a 14-Fr catheter equipped with a downsized SW generator and two polar electrodes at the tip (Fig. 1B). The SW generator had, in principle, an ellipsoidal reflector with a 3.6-mm diameter opening composed of brass and a 0.4- or 0.6-mm core diameter low-hydroxyl content quartz optical fiber whose tip was set as the inner focus point of the reflector. The spherical SWs were generated at a focal point inside a water-filled reflector by irradiations of a Q-switched Holmium (Ho): yttrium aluminum garnet (YAG) laser (SLR-HO-EOQ, Sparkling Photon Inc., Tokyo, Japan) transmitted through an optical fiber. Then, the SWs were refocused at the focal point outside the reflector. We have previously confirmed that ellipticities larger than 1.6 were less preferable for the creation of sharp focusing of the SW [21]. We first developed a reflector with a 1.5 ellipticity and a 2.0-mm focal length and used it for both the epicardial ablation and endocardial ventricular ablation studies. Then, we developed a new reflector with a 1.6 ellipticity and a 3.0-mm focal length and used it for the endocardial AV node ablation study because the deeper focal length was better for targeting the AV node. Direct laser beam irradiation to the cardiac tissue was prevented using shielding film and water-filled spacing between the fiber tip and shielding film (>1.0 mm). Even though the optical fiber in the catheter was broken off, no leakage of the laser beam was noted during the procedure.

### Characteristics of SW Generated by the SWCA System

**Fig 2:**
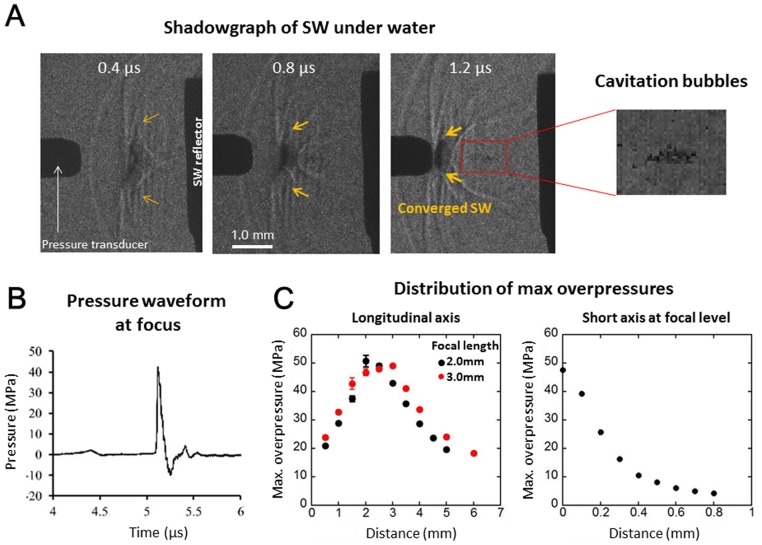
Characteristics of Focused SW Generated by the SWCA System. The shadowgraphs of SW under water taken by high-speed camera showed the converging time course from the reflector onto the focus (0.4, 0.8 and 1.2 μsec). Cavitation bubbles were observed in the third frame (1.2 μsec) (A). The representative pressure waveform of SW at the focus showed a typical time history with instantaneous high positive pressure followed by negative pressure. Max overpressures along the longitudinal axis had a peak at each focal length set by two different reflectors (2.0 and 3.0 mm) (left panel in C), and those across the short axis at the focal level (2.0 mm) were steeply decreased 500 μm in width (right panel in C).

Fig. 2A shows a shadowgraph sequence of the SW emerging from the reflector and converging onto the focus point in the degassed saline. Cavitation bubbles were only observed at the area where the SW passed through when the maximum overpressure of the focused SW exceeded 40 MPa. Fig. 2B shows the typical pressure waveforms of the SW at the focus. It shows a time history of SW with instantaneous high positive pressures followed by negative pressures, which created a tensile force. The laser energy could be finely controlled by changes in the charge voltage of the laser oscillator; there was a positive correlation between the laser energy and the maximum overpressure of the SW (S1 Fig.). The system could induce focused SW with overpressure of up to 50 MPa (60 J/m^2^) at the focus point. The positive pressure distributions along the longitudinal axis in the two different reflectors (2.0 and 3.0 mm) had a peak at each focal range, respectively, and were steeply decreased 500 μm in width across the short axis at the focal region (Fig. 2C). Although peak negative pressure along the longitudinal axis of the ellipsoidal reflector was lowest 0.5 mm short of the focus point, there was a poor correlation between the peak negative pressure and peak positive pressure (S2 Fig.). The standard errors for each measurement of pressure distribution ranged from 0.1 to 2.0.

Preliminary examination showed that the SW application to the femoral muscle and the ventricular myocardium with an epicardial approach caused no temperature rise at the catheter contact site in pigs *in vivo* (S3 Fig.).

### Epicardial Ablation Study in Pigs *in vivo*

First, we examined the pressure threshold that caused myocardial tissue injury by the focused SW (S4 Fig.). No myocardial damage was noted at the sites treated by focused SW under 30 MPa of overpressure on the gross or histopathological examination (0/16 in sham operation and 0/16 in 20–25 MPa). In contrast, we noted some lesions in the RV myocardium only with 30–35 MPa of overpressure (2/16) and noted consistent lesion formation in both ventricular myocardium at all application sites with 40–45 MPa of overpressure (16/16). Based on these results, we determined the energy output of the Ho:YAG laser so that the estimated max overpressure of the focused SW exceeded 40 MPa in the following studies. Next, we examined the necessary duration of SW application to produce myocardial injury. We confirmed that a 30-s SW application was adequate to produce myocardial injury and that the extent of the injury reached a plateau with an application time of 60 s with consistent formation of spheroidal lesions (S5 Fig.).

**Fig 3:**
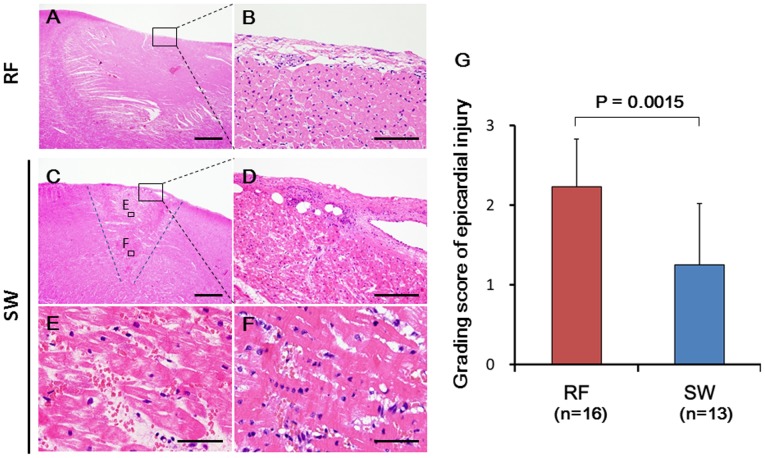
Histopathological Findings of Epicardial Lesions in the Acute Phase. The RF lesions were semi-circular in shape (A) with severe superficial tissue damages including the thinning of adventitial membrane (B; enlarged view of the black square in E). The SW-induced lesions were spheroidal in shape toward the focus (the blue dash line in C) with mild superficial tissue damage (D), disruptions of myocardial fibers and interstitial hemorrhage beyond the focus (E; enlarged view of the black square in C), and the strongest myocardial tissue degeneration including contraction band necrosis at the focus (F; enlarged view of the black square in C). The histological grading scores of epicardial injury were significantly different between the RF- and SW-induced lesions (G). The specimens were stained with hematoxylin—eosin. Scale bars: 1.0 mm (A and E), 200 μm (B and F), and 50 μm (C and D). The results are expressed as mean ± standard deviation (SD). The Mann—Whitney’s U test was used to compare the histological grading score between the SW and RF lesions.

In the acute phase, the SW application sites macroscopically appeared as circular dark violet lesions that were in the shape of the end face of the SW reflector. Histopathological examination showed spheroidal lesions, including disruptions of myocardial fibers with interstitial hemorrhage toward the SW focus (Fig. 3C and E). Importantly, the strongest myocardial tissue degeneration was noted at the SW focus site, where eosinophilic change of the myocardial cell body and contraction band necrosis were noted (Fig. 3F). In contrast, severe tissue damage starting from the catheter contact surface was noted in the RF lesions (Fig. 3B), whereas the superficial tissue damage was mild, and the adventitial cells were relatively maintained in the SW lesions even after an 180-s application (Fig. 3D). The histological grading score for epicardial injury was significantly less in the SW lesions compared with the RF lesions (Fig. 3G).

**Fig 4:**
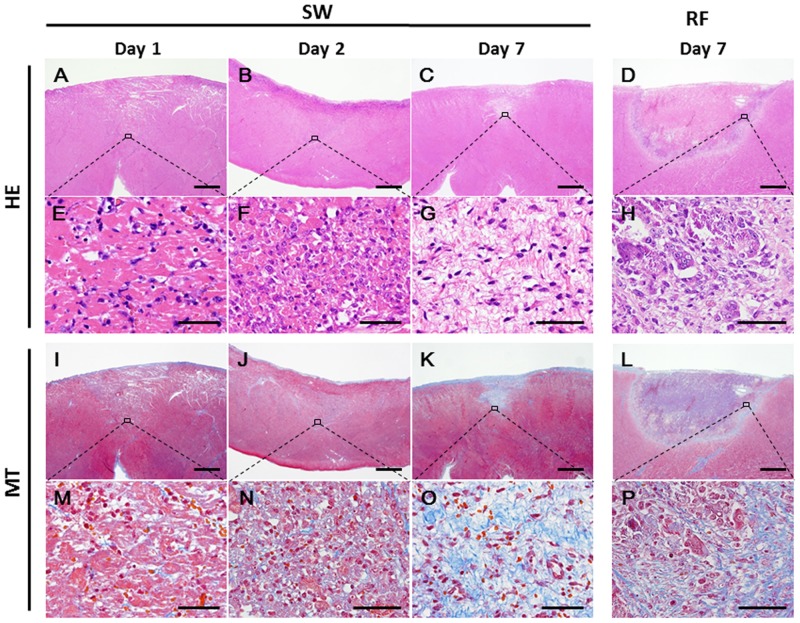
Time-course of Histopathological Findings of Epicardial Lesions. The SW-induced lesion at Day 1 (A, E, I, and M), Day 2 (B, F, J, and N), and Day 7 (C, G, K, and O) showed the sphenoidal lesions in each phase. The magnified images at the SW focus site showed the infiltration of inflammatory cells around the degenerated myocardium at Day 1 (E) and more at Day 2 (F). The SW-induced lesion showed homogeneous fibrotic changes at Day 7 (K and O). The RF lesion showed central residual necrotic tissue (D) and a border zone with fibrosis (L and P) and chronic infiltration of inflammatory cells (H) at Day 7. The specimens were stained with hematoxylin—eosin (HE) (A–H) and Masson’s trichrome (MT) (I–P). Scale bars: 1.0 mm (A–D and I–L), and 50 μm (E–H and M–P).

Next, we performed the survival study to examine the time course of SW-induced myocardial lesions (Fig. 4). Histological examination showed the spheroidal shape of the myocardial lesions with the infiltration of neutrophils and mononuclear cells around the degenerated myocardium at Day 1 and to a greater extent at Day 2. In addition, the homogenous fibrotic lesions were noted at Day 7. In contrast, the RF lesions at Day 7 were characterized by residual central necrotic tissue and a fibrotic border zone with chronic infiltration of inflammatory cells.

**Fig 5:**
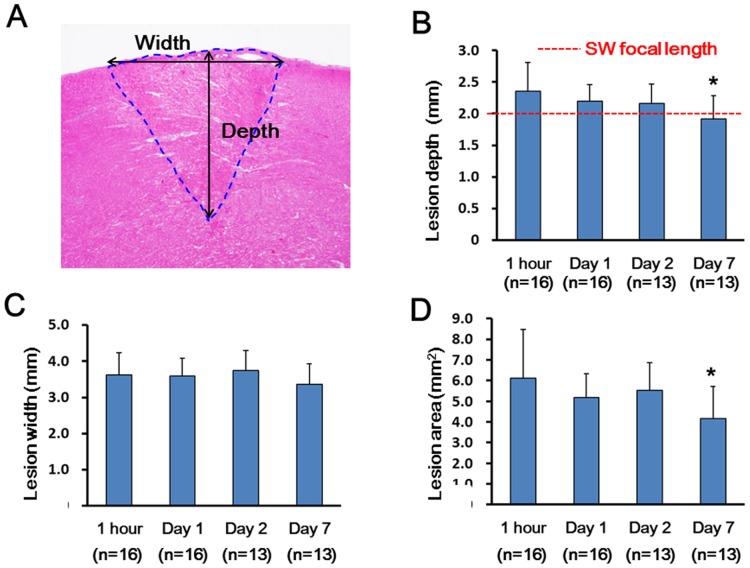
Time course of Depth, Width and Area of the SW-induced Lesions. The lesion depth and width were measured as indicated by the arrows, and the area was measured as indicated by the dashed line (A). The lesion depth was maximal at 1 h after the procedure and decreased over time with the depth equivalent to the SW focal length (2.0 mm; the red dashed line) at Day 7 (B). The lesion width was equivalent to the opening diameter of the SW reflector (3.6 mm) (C). The lesion area was also maximal at 1 h and decreased over time (D). Results are expressed as mean ± SD. One-way analysis of variance was used to compare data for statistically significant differences, followed by the Tukey’s honestly significant difference (HSD) to elucidate any interactions among the lesion depth, width, and area at each time course. *P < 0.05 vs. 1 h.

The lesion depth at each phase (1 h, Day 1, Day 2, and Day 7) for the SW lesions was 2.36 ± 0.45 mm (n = 16), 2.20 ± 0.27 mm (n = 16), 2.16 ± 0.26 mm (n = 13), and 1.91 ± 0.37 mm (n = 13), respectively. Despite the decrease in depth over time (1 h vs. Day 7, P = 0.008), the lesion depth was fairly comparable to the SW reflector’s focal length (2.0 mm) even at Day 7 (Fig. 5B). The lesion width was maximal at the surface (3.62 ± 0.61 mm at 1 h, n = 16) and comparable to the opening diameter of the SW reflector (3.6 mm) (Fig. 5C); the width was gradually decreased toward the focus. The lesion area was decreased over time (1 h vs. Day 7, P = 0.015) similar to the decrease in depth, but was fairly maintained even at Day 7 (Fig. 5D). The lesions were similarly distributed in a spheroidal shape, and the depth, width and area were similar in both ventricles (S6 Fig.).

In the epicardial ablation study, no fatal adverse effects were noted during the procedures, such as hemorrhage, cardiac rupture, or malignant VAs.

### Endocardial Ventricular Ablation Study in Pigs *in vivo*

We examined whether the endocardial SW application to the ventricular wall could also cause myocardial lesions. The result showed that the focused SW application to the RV myocardium with the endocardial approach also caused spheroidal lesions similarly to the epicardial lesions (S7D Fig.). Although endothelial damage and micro-thrombus formation were also noted with the SW lesions, those changes were different from those in the RF lesions. Massive endothelial damage was noted in the RF lesions, whereas only partial detachment of the endothelial cells was noted in SW lesions (S7 Fig.). The depth of the RF-induced lesions was 4.97 ± 1.05 mm, which was comparable to that of the previous report [22]. On the other hand, the depth of the SW-induced lesion was 2.11 ± 0.45 mm (n = 6), which was comparable to the SW focal length (S8 Fig.).

### Endocardial AV Node Ablation Study in Pigs *in vivo*

**Fig 6:**
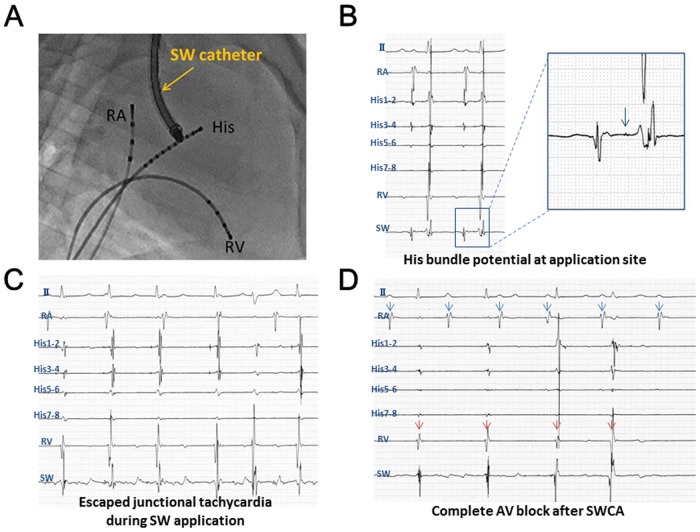
AV Node Ablation by the SWCA System. The SW catheter was inserted through the jugular vein approach (A) and located at the target site where the His-bundle potential (arrow) was recognized (B). A junctional escape rhythm was observed immediately after starting the SW application (C), followed by a complete AV block (D). Blue and red arrows show the atrial and ventricular potentials, respectively.

**Fig 7:**
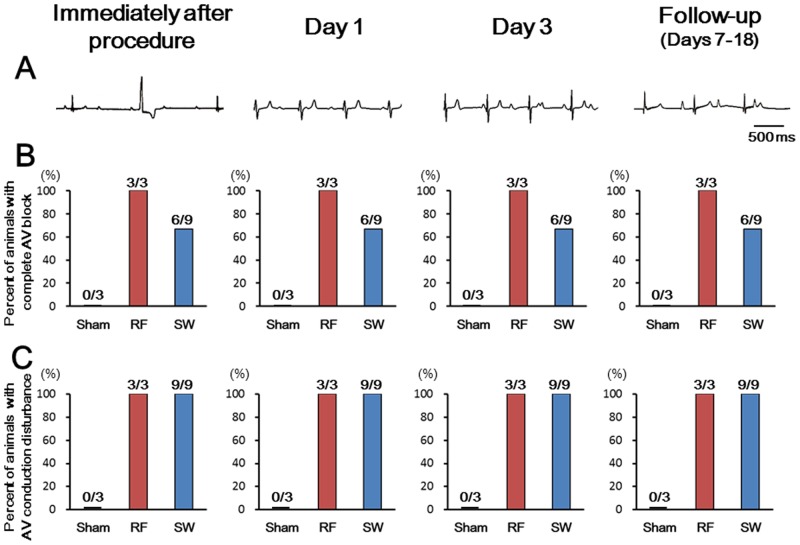
Persistent Electrophysiological Effect of SWCA. Representative ECGs with AV dissociation in a SW-treated animal (A) and the percentage of animals presenting with complete AV block (B) or any AV conduction disturbance including complete AVB in panel B (C) immediately after procedure, and at Day 1, Day 3, and at the end of the follow-up period. The data from Day 1 and Day 3 were obtained from the Holter electrocardiogram with pacing (VVI 80 beats/min). The AV conduction disturbance in both RFCA and SWCA were sustained during each follow-up period. The number on the top of the bar graphs show the animals with complete AV block (B) and those with AV conduction disturbance (C) to the total animal numbers (Sham, n = 3; RFCA, n = 3; SWCA, n = 9).

We examined whether the endocardial SW application to the AV node could cause electrophysiological effects in a normal pig. In the sham-operated animals, no electrophysiological change was noted in both the acute study (n = 3) and in the survival study (n = 3; 11.7 ± 2.1 days). In the RF-treated animals, complete AV block was successfully achieved in all animals (n = 6) and persisted for 11.7 ± 2.1 days in all surviving animals (n = 3) (S2 Table). In the SW-treated pigs, the SWCA caused AV nodal electrophysiological property changes in all animals (n = 14) immediately after the procedure, including junctional escape rhythms, AV conduction delays and AV dissociation (S2 Table). Consequently, an AV block was achieved in 12 of 14 SW-treated animals (complete AV block in 9 of 12), whereas in the remaining 2 animals, a junctional escape rhythm was induced. Fig. 6 shows the representative ECG changes in a SW-treated pig (Pig SW7 in S2 Table), in which the junctional escape rhythm was noted immediately after starting the SW application to the AV node; this was followed by a complete AV block. In the survival study, the electrophysiological effects of SWCA persisted for 12.6 ± 3.9 days in all surviving SW-treated animals (n = 9) (Fig. 7, S2 Table). The complete AV block persisted for the entire follow-up period in 6 of 9 animals; however, in the remaining 3, the advanced AV block continued until Day 8 in 1 animal and gradually improved to a first-degree AV block at Day 14 in the other 2.

In the SWCA group, no fatal adverse effects such as cardiac rupture or malignant VAs were noted during the procedures,. No animals experienced sudden death during the follow-up period. The Holter electrocardiogram for first 3 days after the procedure showed no malignant arrhythmias. Echocardiography showed no pericardial effusion before euthanasia in all animals.

**Fig 8:**
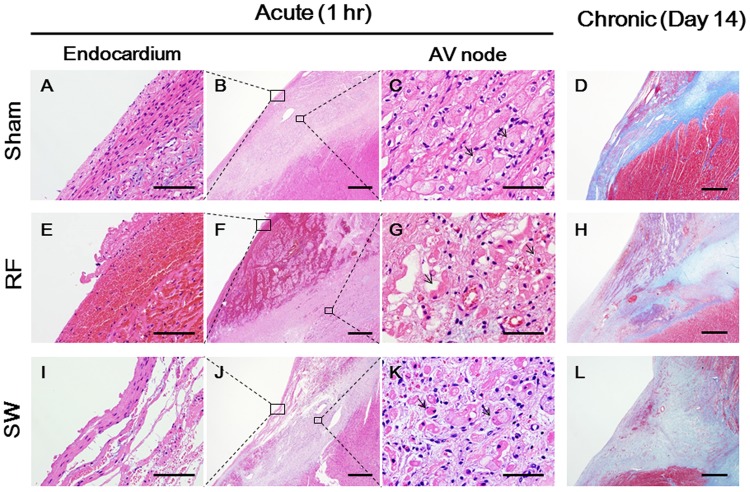
Histopathological Examination of the AV Node after Ablation. In the sham operated group (A–D), no morphological change of AV nodal cells (black arrows in C) was noted except for slight interstitial fibrosis (D). In the RFCA group (E–H), the thermal degeneration of AV nodal cells (black arrows in G) in the acute phase and fibrotic lesions with residual central necrosis in the chronic phase (H, day 14) were noted. Massive endothelial damage was also noted (E). In the SWCA group (I–L), the degeneration of AV nodal cells, including cell body atrophy (black arrows in K) in the acute phase and homogenous fibrotic lesions in the chronic phase (L, day 14), were noted.The specimens were stained with hematoxylin—eosin (A–C, E–G and I–K) and Masson’s trichrome (D, H and L). Scale bars: 1.0 mm (panels D, H, and L), 500 μm (B, F, and J), 100 μm (A, E, and I), and 50 μm (C, G, and K).

In the sham group, there was no histopathological change in the AV node except for mild endocardial fibrosis probably due to mechanical damage of the SW catheter tip (Fig. 8A–D). Histopathological examination showed the degeneration of the AV nodal cells, including cell body atrophy in the acute phase in the SWCA group (Fig. 8I and 8K). In the survival study, the SW-treated site showed homogenous fibrotic lesions, which did not have the structure of the AV node at Day 14 (Fig. 8L). Similar to the endocardial ablation study on the RV, microthrombus formation was noted in both the SWCA and RFCA groups. However, endothelial damages were less in the SWCA than in the RFCA group (S9 Fig.).

## Discussion

The major findings of the present study were the following points: (1) we were able to develop a novel SWCA system, (2) the SWCA system could cause persistent myocardial lesions characterized by less superficial injury and at a depth according to the focal length, (3) the SWCA system could cause sustained AV conduction disturbances with an endocardial approach, and (4) the SWCA system had an acceptable safety level without fatal adverse effects in pigs *in vivo*. Thus, our novel SWCA system may be able to reduce the risk of thrombogenesis and create deeper lesions than the RFCA if a deeper SW focal length is achieved in future studies.

### Mechanisms of Myocardial Tissue Damage Caused by Focused SW

In the present study, we successfully miniaturized the mechanisms of generating focused SW with an overpressure that was compatible to that used in ESWL. To the best of our knowledge, this is the first report that demonstrated the detailed characteristics of myocardial lesions caused by a focused SW application. It is believed that SW causes tissue injury through the combination of two different mechanical stresses, including shear force and the cavitation effect [23]. The pressure threshold that caused tissue injury has been reported to be 3–19 MPa in the kidney [24], 2–10 MPa in the lung [25], and 1 MPa in the brain [26]. In the present study, the overpressure that stably caused ventricular myocardial damage was over 40 MPa, which was higher than that required by other organs [24–26]. This may be due to the difference in acoustic impedance depending on the cellular structure among each organ and the smaller number of SW applications in this study (180 shots) compared with the previous studies (1000–1500 shots) [24], [25]. When the max overpressure of SW exceeded 40 MPa at the focal site, myocardial injury was also noted at the prefocal zone, where the overpressure was below 30 MPa. On the other hand, focused SW under 30 MPa caused no tissue injuries even at the focal site. These results indicated that myocardial injury at the prefocal zone was caused not only by a compression effect but also by other effects, such as the cavitation effect judging from the basic study in the degassed saline.

### Characteristics of Non-thermal Myocardial Lesions by SW Application

The RFCA caused myocardial lesions through thermal coagulation necrosis; these were associated with persistent chronic inflammation, surrounding fibrosis, and varying degrees of residual central myocyte necrosis that depended on the age of the lesion as observed in the present study [7]. In contrast, the SWCA-induced lesions had different characteristics from thermal lesions by the RF. It has been reported that ESWL-induced renal injury triggered the infiltration of inflammatory cells, leading to scar formation with fibrosis, calcification, and permanent loss of functional renal tissues [27]. In the present study, focused SW induced similar myocardial conditions as ESWL, where myocardial disruption with interstitial hemorrhage and contraction band necrosis initially occurred, followed by the infiltration of inflammatory cells with a resultant formation of homogenous fibrotic lesions at Day 7. This time course of irreversible myocardial cell death proceeding to persistent fibrosis was suitable for ablation lesions in the treatment of arrhythmias. These results suggested that the SWCA system had the favorable characteristics with shorter-term inflammation and early fixation of lesions as a non-thermal ablation modality. In addition, the heat energy from the RFCA was occasionally limited due to a rise in temperature and an impedance drop to prevent steam pops [28]. However, the non-thermal SWCA could always deliver the required energy for SW because of the lack of the risk of steam pops, which resulted in stable lesion formation in any conditions.

### Advantages of the SWCA System

The dimensions of the RF lesion began at the contact area and decreased proportionally depending on the distance, resulting in superficial tissue damages and a limited lesion depth in thick myocardium [7], [8]. This could be the reason for the incomplete treatment of deep arrhythmic foci and the thrombus formation. In the past, efforts have been made to improve the depth of ablation therapy. An irrigated RF catheter is one of the examples [29], [30]; however, the depth of an RF lesion may not be adequate to treat any type of VTs of epicardial origin when the endocardial approach was used [10], [11]. Furthermore, some arrhythmogenic substrates may exist deep in the intramural tissue beyond the reach of the combination of endo- and epicardial ablation [12], [13]. Bipolar ablation and transcoronary ethanol ablation have been developed for such substrates; however, the technical difficulty and higher risk of complications have restricted those procedures only to a selected population at high-volume centers [31], [32]. A catheter ablation system using high-intensity focused ultrasound (HIFU) has been developed and is a clinically useful modality that utilizes an alternative energy source [33], [34]. This system could focus the energy on the mid-layer of the myocardium; however, the main mechanism of tissue injury is coagulation necrosis by heating as in the RF [33], [34]. In the present study, we demonstrated that our non-thermal SWCA system could cause myocardial lesions with a depth according to the focal length, although the selection of depth still needs to be improved. In addition, the strongest myocardial degeneration was noted at the focus site, and the damage at the superficial site was less compared with the RFCA. This could be one of the advantages of the SWCA in the reduction in the risk of thrombus formation. Cryoablation is also an advanced modality that could reduce thrombus formation [35]. However, permanent injury formation usually requires prolonged freezes for approximately 4 min [35]. Thus, the SWCA system with a shorter application time may have an advantage over cryoablation in this regard.

### Effectiveness and Safety of the SWCA System

We demonstrated the feasible electrophysiological effects of our SWCA system in the AV node ablation experiment. These results suggested that the focused SW could also damage the specialized cardiac muscle cells of the conduction system in addition to the normal myocardial tissues. Importantly, we observed no fatal complications in the acute or long-term studies. The novel SWCA system is safe because the lesion dimension is easily controlled according to focal length. In addition, the characteristics of SW as a non-thermal energy may be helpful to avoid the side effects associated with thermal ablation such as steam pops [28].

### Study Limitations

Several limitations should be mentioned for the present study. First, the SWCA system used in the present study was a prototype model equipped with reflectors of a relatively short focal length (2.0–3.0 mm). Thus, the lesion depth with the present SWCA system was not comparable to the RFCA. This may have caused the difference in the achieved AV nodal conduction disturbances among the SWCA-treated animals. In addition, in the present study, mechanical endothelial tissue damage, but not thermal damage, was induced with a resultant thrombus formation during the endocardial ablation study because the superficial site was also exposed to SW with overpressures exceeding 20 MPa. The desired lesion depth that would be needed to ablate all arrhythmic foci, including epicardial and deep ventricular septal foci; in the endocardial approach, this would be approximately 10 mm. We are presently developing a folding reflector with a larger opening diameter and a longer focal length to achieve a deeper lesion and to further reduce superficial injury. Second, the SW-induced myocardial lesion had gradually regressed over time. Thus, the time course of the SW-induced lesions dose need to be fully clarified. The regression phenomenon may be due to absorption of the swollen lesion caused in the acute phase. Third, even though the AV block was created using the SWCA system in the endocardial AV node ablation study, the conduction block was not systematically analyzed. Fourth, because the SW application was not synchronized with heartbeats in the present study, extrasystoles in response to the SW were noted in some animals. Although we observed no VTs triggered by R-on-T extrasystole, the synchronization function should be equipped in the SWCA system to achieve a stable and anticipated lesion depth. Fifth, we examined the effect of the focused SW on the heart only in normal pigs. Patients with cardiac diseases have inhomogeneous acoustic impedance due to the mixture of normal myocardium, fibrosis, and dense scars, and the conduction of SW could be different in abnormal myocardium with inhomogeneous acoustic impedance. Thus, it remains to be examined whether the focused SW could cause similar lesions in abnormal cardiac tissue such as infarcted myocardium. Sixth, the RV myocardium was more likely to be damaged by SW than the LV (S4 Fig.). We assumed that the difference in tissue acoustic impedance between both ventricles was one of the reasons because the RV myocardial wall is softer than LV, particularly in the systolic phase. However, the detailed mechanisms remain to be explained. Seventh, the mechanism of myocardial tissue injury has not been fully elucidated. Because it was difficult to completely exclude the generation of cavitation bubbles *in vivo*, we were unable to separate the cavitation effect and compression effect on the tissue injury. Finally, the further long-term safety of the SWCA remains to be examined in future clinical studies.

### Conclusions

We were able to develop a novel, non-thermal catheter ablation system using focused SW as an energy source, demonstrating the effectiveness and safety of the SWCA system in pigs *in vivo*. The proposed novel SWCA system may be a promising option to compensate for the weaknesses of the current RFCA therapy. However, the novel SWCA system still needs improvements as the current prototype showed a shallower depth than the RF and the presence of micro-thrombus formation in animal studies.

## Supporting Information

S1 Fig Correlation between Laser Energy and SW Amplitude. The Ho:YAG laser energy could be controlled by changes in the charge voltage of the laser oscillator (**A**). There was a positive correlation between laser energy and the maximum overpressure of SW (**B**).
(TIF)

S2 Fig Correlation between Peak Positive Pressure and Peak Negative Pressure of SW. The peak negative pressure along the longitudinal axis of the SW reflector was lowest 0.5 mm short of the focus point (**A**). There was a poor correlation between peak negative pressure and peak positive pressure (**B**).
(TIF)

S3 Fig Surface Temperature Change of Thigh Muscle and Ventricular Myocardium during SW Application in Pigs *in vivo*. The focused SW was applied to the thigh muscle and ventricular myocardium with epicardial approach, and surface temperature just below the catheter was continuously measured for 3 min in pigs *in vivo* (n = 3). There was no temperature rise over 50°C that could cause thermal tissue necrosis.
(TIF)

S4 Fig Threshold Pressure of Myocardial Damage by SWCA. The focused SW was applied to ventricular myocardium in four different energy output estimating 0 MPa (Sham), 20–25 MPa, 30–35 MPa and 40–45 MPa. The left panel shows the percent of the lesions confirmed in the right ventricle at each overpressure. Right panel shows the percent of lesions in the left ventricle. The confirmed lesion was defined as the presence of histopathological changes, including myocardial tissue disruption, interstitial hemorrhage and contraction band necrosis. The myocardial lesions were noted only in the right ventricular myocardium under 30–35 MPa of overpressure and at all application sites under 40–45MPa of overpressure.
(TIF)

S5 Fig Correlation between Lesion Formation and Duration of SW Application. The focused SW was applied to ventricular myocardium for three different durations (30, 60, and 120 s) by 1 Hz. Partial myocardial injury was confirmed at the SW focal site even after a 30-s application (the blue dashed circle). The spheroidal lesions were consistently created by SW application for over 60 s (the blue dashed line). Panel A shows the histopathological findings. The specimens were stained with hematoxylin—eosin, and the scale bars represent 1.0 mm. Panel B shows the percent of the lesions confirmed in the right ventricle or the left ventricle.
(TIF)

S6 Fig Comparison of Lesion Distribution between the Right Ventricle and the Left Ventricle. The histopathological specimens showed the epicardial SW-induced lesions in the right ventricle (RV) and the left ventricle (LV). The lesion depth, width and area were similar in both ventricles (n = 6 in the RV and n = 10 in the LV). The specimens were stained with hematoxylin—eosin, and the scale bars represent 1.0 mm. Results are expressed as mean ± SD. The Student’s t-test was used to compare the depth, width, and area between the RV and LV lesions.
(TIF)

S7 Fig Histopathological Findings of Right Ventricular Endocardial Lesions in the Acute Phase. The RF lesion was semi-circular in shape (A) with the loss of endothelial membrane (B and C; enlarged view of the black square in A). The endocardial SW-induced lesion was spheroidal in shape as in the epicardial study with mild endothelial damages (E and F; enlarged view of the black square in D). Histological grading scores of the endothelial injury were significantly different between the SW- and RF-induced lesions (G).The specimens were stained with hematoxylin—eosin (A, B, D, and E) or Elastica—Masson (C and F). Scale bars: 1.0 mm (A and D), and 200 μm (B, C, E, and F). Results are expressed as mean ± SD. The Mann—Whitney’s U test was used to compare the histological grading score between the SW and RF lesions.
(TIF)

S8 Fig Comparison of Lesion Distribution between Epicardial Ablation and Endocardial Ablation. The upper panels show the RF-induced lesions from either epicardial or endocardial ablation. The lesion depth, width, and area were similar in both approaches (n = 13 with epicardial ablation and n = 6 with endocardial ablation). The lower panels show the SW-induced lesions. The lesion distribution was similar in both approaches (n = 16 with epicardial ablation and n = 6 with endocardial ablation). The specimens were stained with hematoxylin—eosin, and the scale bars represent 1.0 mm. Results are expressed as mean ± SD. The Student’s t-test was used to compare the depth, width, and area between the epicardial and endocardial lesions.
(TIF)

S9 Fig Histopathological Findings of Endothelial Injury and Thrombus Formation in the Endocardial Ablation Experiment. The endothelial damage characterized by a massive loss of endothelium in the RFCA group (A and B) and partial detachment in the SWCA group (C and D) was observed with micro-thrombus formation in both groups. Histological grading scores of endothelial injury were significantly different between the RFCA group and SWCA group (E). The specimens were stained with Elastica-Masson. Scale bars represent 1.0 mm in panels A and C, and 200 μm in panels B and D. Results are expressed as mean ± SD. The Mann—Whitney’s U test was used to compare the histological grading score between the SW and RF lesions.
(TIF)

S1 Table (DOCX)

S2 Table (DOCX)
