# Supplementary figures and images for: Development of a Novel Shock Wave Catheter Ablation System -The First Feasibility Study in Pigs-
Source: PLoS One. 2015 Jan 29;10(1):e0116017. doi: 10.1371/journal.pone.0116017 (PMC4310588; doi:10.1371/journal.pone.0116017)

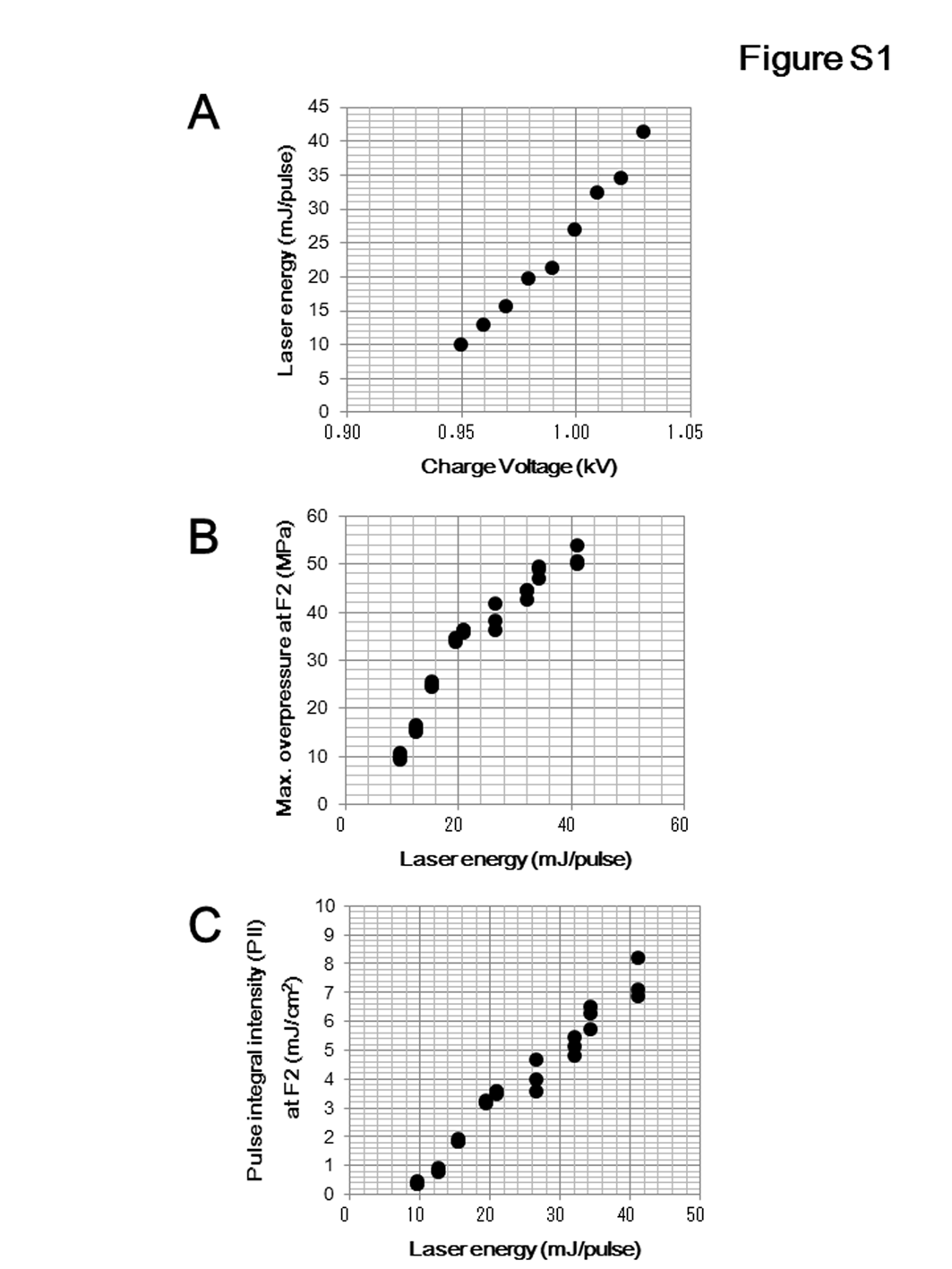

Supplement: S1 Fig — The Ho:YAG laser energy could be controlled by changes in the charge voltage of the laser oscillator (A). There was a positive correlation between laser energy and the maximum overpressure of SW (B). (TIF) [file pone.0116017.s001.tif]

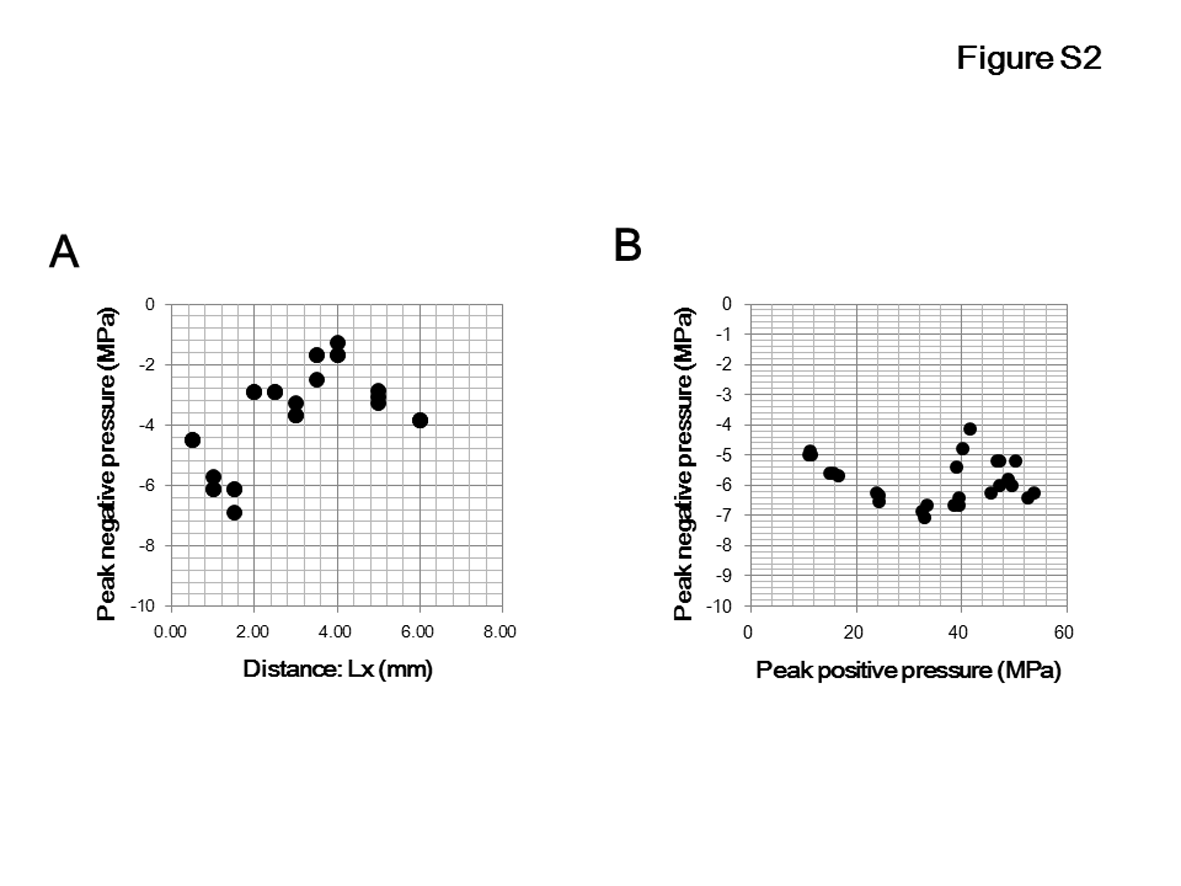

Supplement: S2 Fig — The peak negative pressure along the longitudinal axis of the SW reflector was lowest 0.5 mm short of the focus point (A). There was a poor correlation between peak negative pressure and peak positive pressure (B). (TIF) [file pone.0116017.s002.tif]

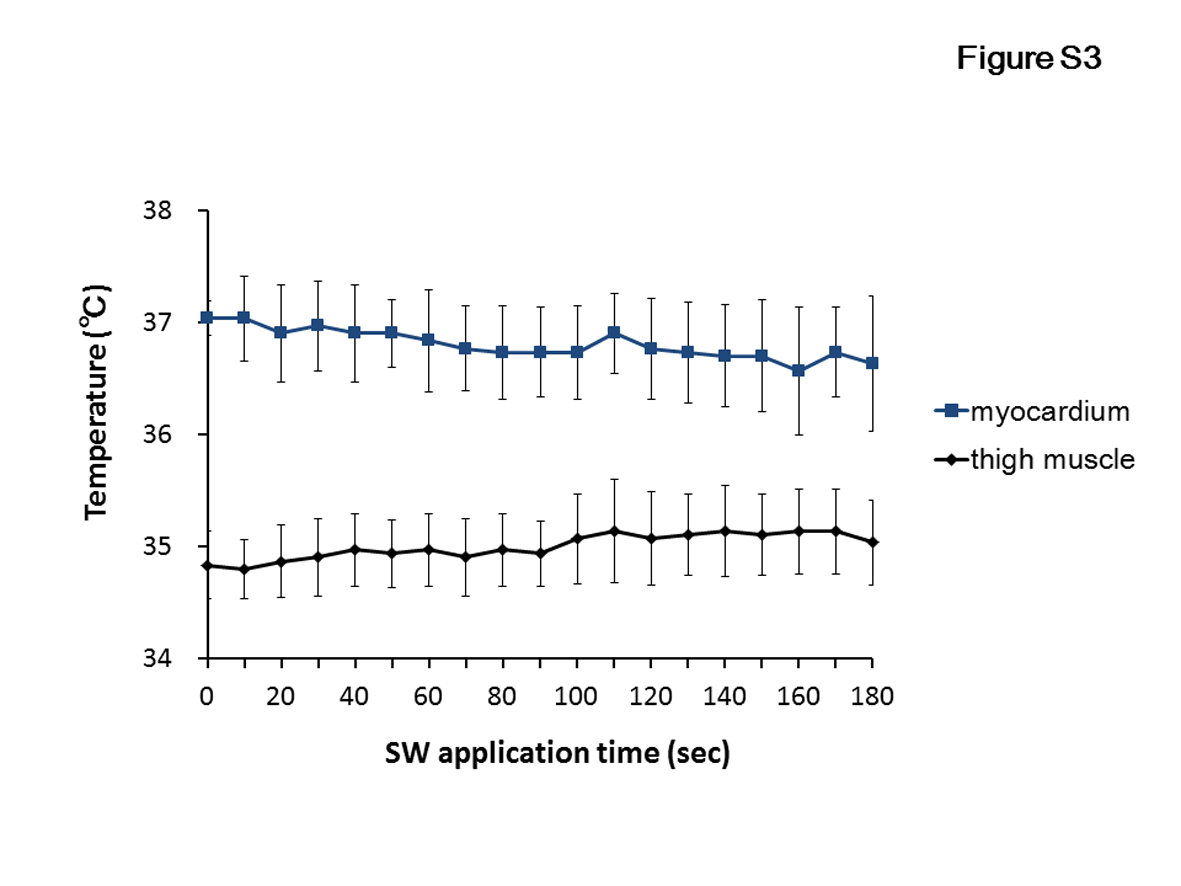

Supplement: S3 Fig — The focused SW was applied to the thigh muscle and ventricular myocardium with epicardial approach, and surface temperature just below the catheter was continuously measured for 3 min in pigs in vivo (n = 3). There was no temperature rise over 50°C that could cause thermal tissue necrosis. (TIF) [file pone.0116017.s003.tif]

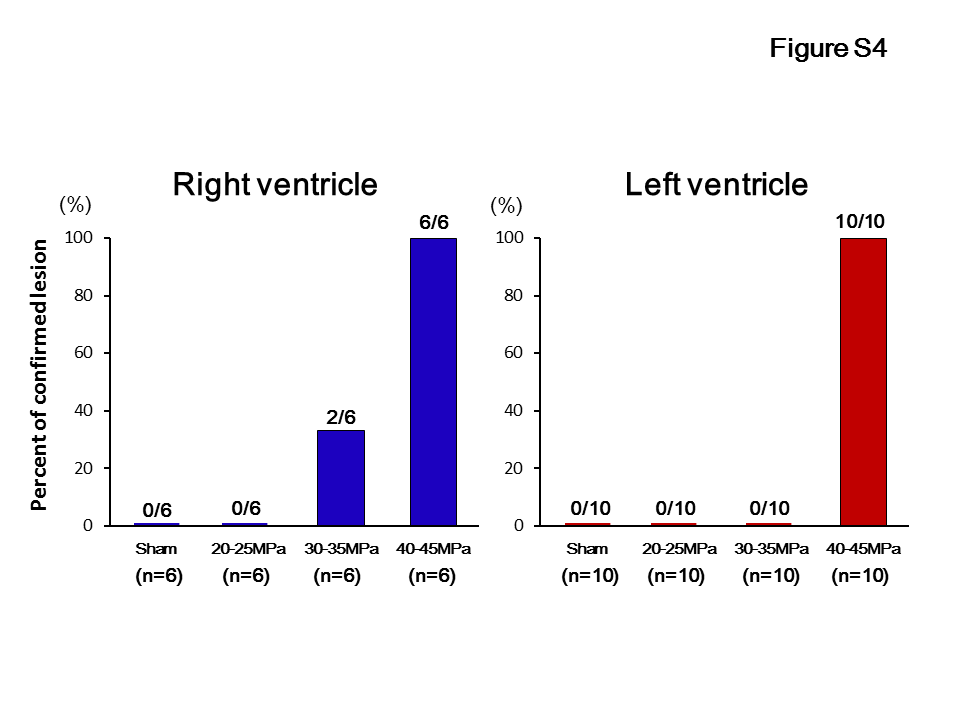

Supplement: S4 Fig — The focused SW was applied to ventricular myocardium in four different energy output estimating 0 MPa (Sham), 20–25 MPa, 30–35 MPa and 40–45 MPa. The left panel shows the percent of the lesions confirmed in the right ventricle at each overpressure. Right panel shows the percent of lesions in the left ventricle. The confirmed lesion was defined as the presence of histopathological changes, including myocardial tissue disruption, interstitial hemorrhage and contraction band necrosis. The myocardial lesions were noted only in the right ventricular myocardium under 30–35 MPa of overpressure and at all application sites under 40–45MPa of overpressure. (TIF) [file pone.0116017.s004.tif]

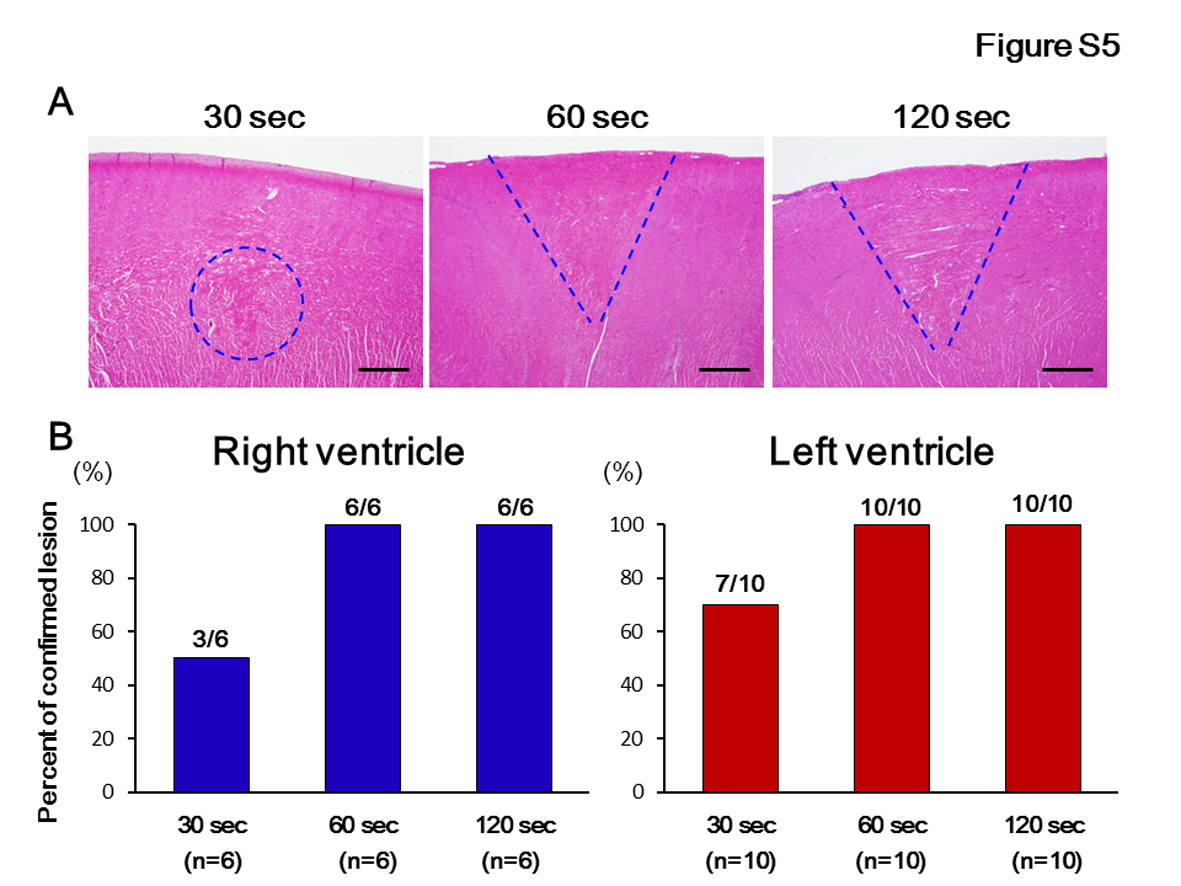

Supplement: S5 Fig — The focused SW was applied to ventricular myocardium for three different durations (30, 60, and 120 s) by 1 Hz. Partial myocardial injury was confirmed at the SW focal site even after a 30-s application (the blue dashed circle). The spheroidal lesions were consistently created by SW application for over 60 s (the blue dashed line). Panel A shows the histopathological findings. The specimens were stained with hematoxylin—eosin, and the scale bars represent 1.0 mm. Panel B shows the percent of the lesions confirmed in the right ventricle or the left ventricle. (TIF) [file pone.0116017.s005.tif]

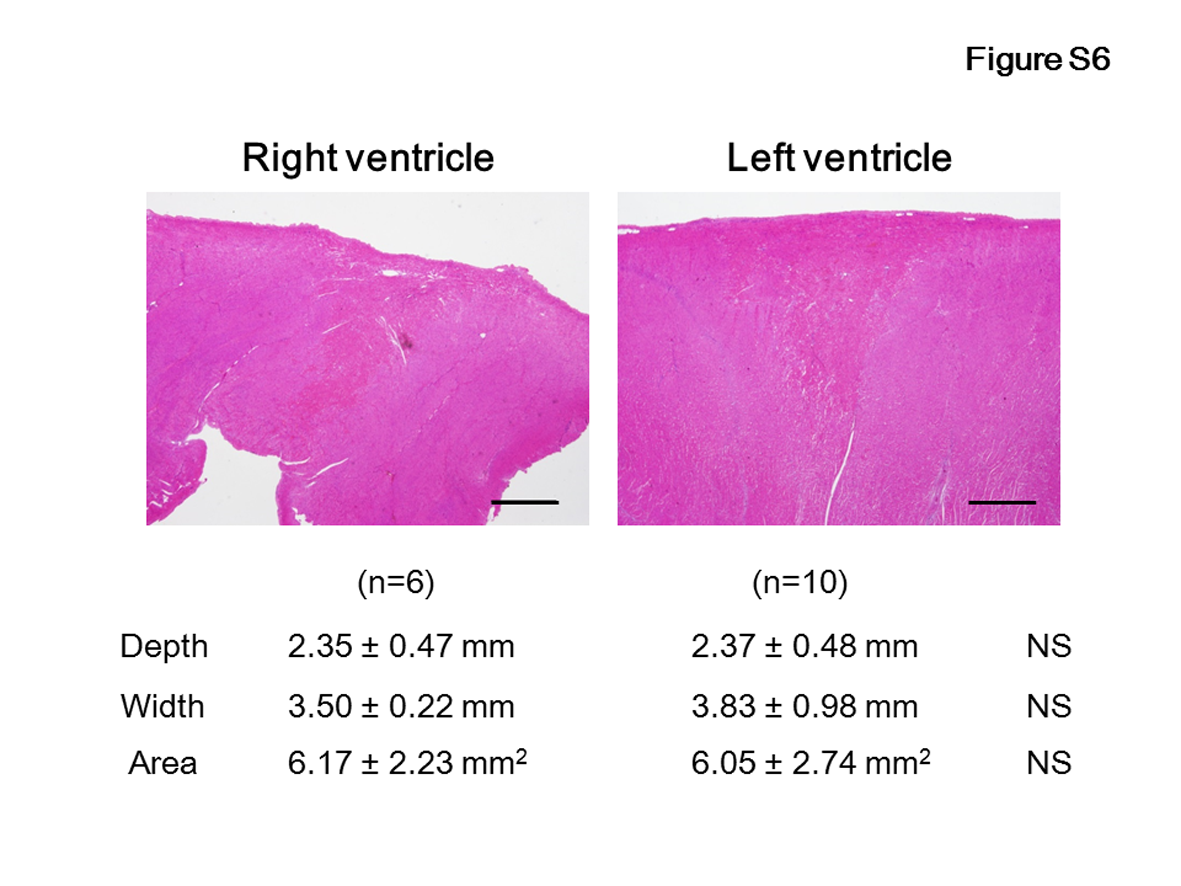

Supplement: S6 Fig — The histopathological specimens showed the epicardial SW-induced lesions in the right ventricle (RV) and the left ventricle (LV). The lesion depth, width and area were similar in both ventricles (n = 6 in the RV and n = 10 in the LV). The specimens were stained with hematoxylin—eosin, and the scale bars represent 1.0 mm. Results are expressed as mean ± SD. The Student’s t-test was used to compare the depth, width, and area between the RV and LV lesions. (TIF) [file pone.0116017.s006.tif]

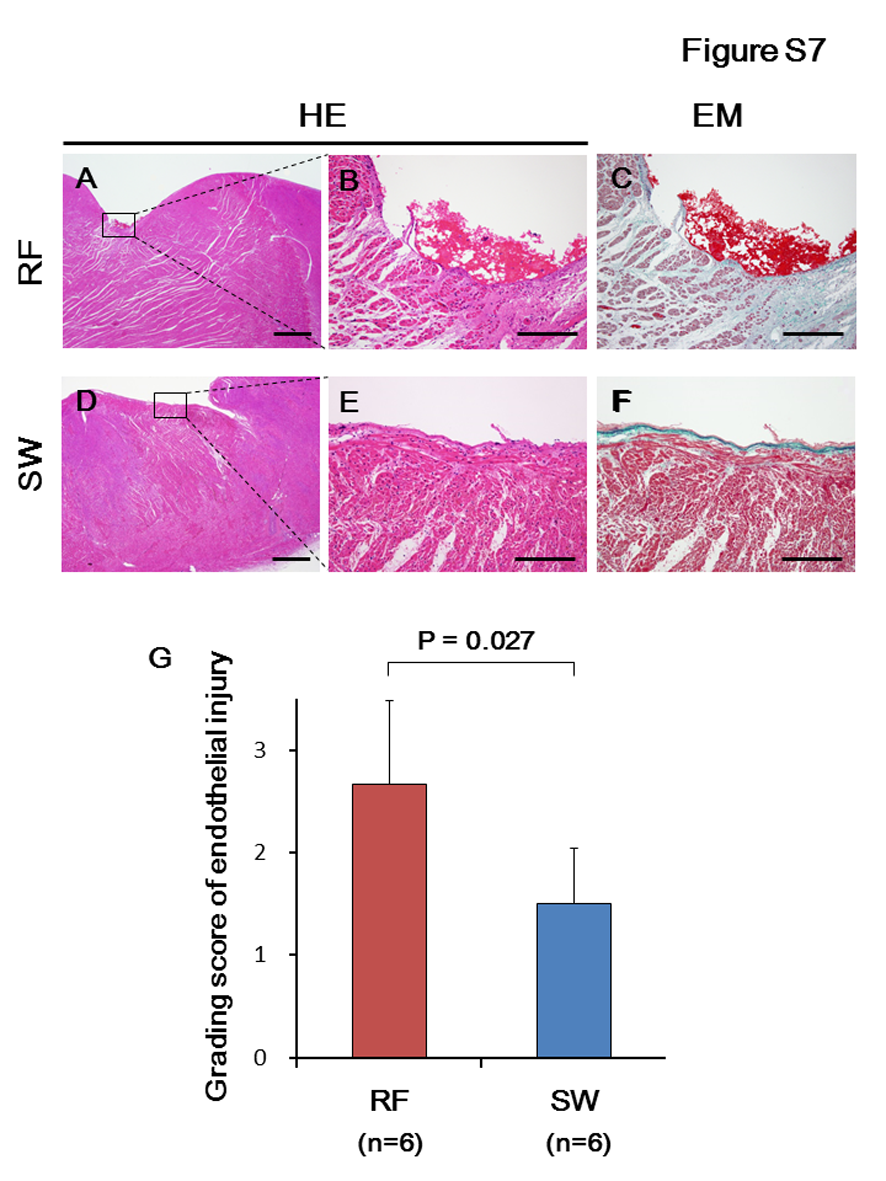

Supplement: S7 Fig — The RF lesion was semi-circular in shape (A) with the loss of endothelial membrane (B and C; enlarged view of the black square in A). The endocardial SW-induced lesion was spheroidal in shape as in the epicardial study with mild endothelial damages (E and F; enlarged view of the black square in D). Histological grading scores of the endothelial injury were significantly different between the SW- and RF-induced lesions (G).The specimens were stained with hematoxylin—eosin (A, B, D, and E) or Elastica—Masson (C and F). Scale bars: 1.0 mm (A and D), and 200 μm (B, C, E, and F). Results are expressed as mean ± SD. The Mann—Whitney’s U test was used to compare the histological grading score between the SW and RF lesions. (TIF) [file pone.0116017.s007.tif]

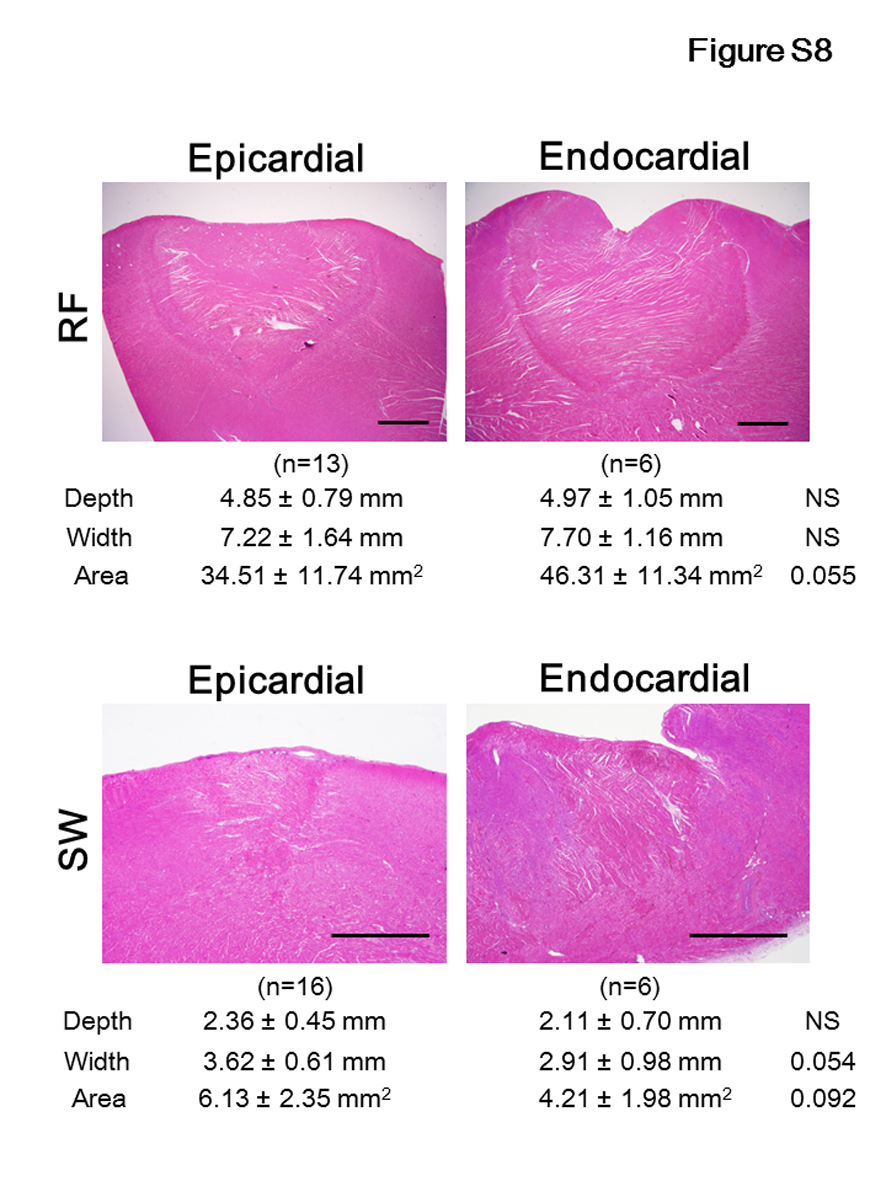

Supplement: S8 Fig — The upper panels show the RF-induced lesions from either epicardial or endocardial ablation. The lesion depth, width, and area were similar in both approaches (n = 13 with epicardial ablation and n = 6 with endocardial ablation). The lower panels show the SW-induced lesions. The lesion distribution was similar in both approaches (n = 16 with epicardial ablation and n = 6 with endocardial ablation). The specimens were stained with hematoxylin—eosin, and the scale bars represent 1.0 mm. Results are expressed as mean ± SD. The Student’s t-test was used to compare the depth, width, and area between the epicardial and endocardial lesions. (TIF) [file pone.0116017.s008.tif]

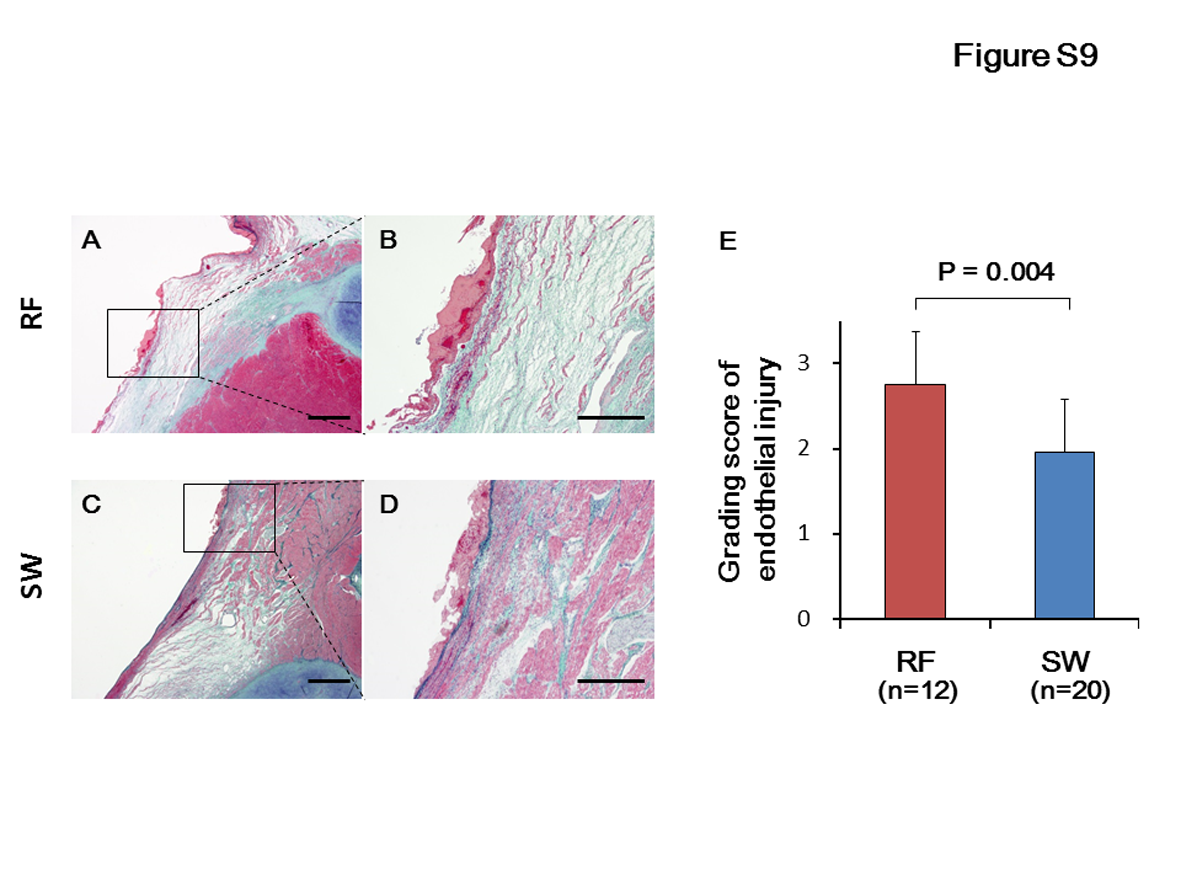

Supplement: S9 Fig — The endothelial damage characterized by a massive loss of endothelium in the RFCA group (A and B) and partial detachment in the SWCA group (C and D) was observed with micro-thrombus formation in both groups. Histological grading scores of endothelial injury were significantly different between the RFCA group and SWCA group (E). The specimens were stained with Elastica-Masson. Scale bars represent 1.0 mm in panels A and C, and 200 μm in panels B and D. Results are expressed as mean ± SD. The Mann—Whitney’s U test was used to compare the histological grading score between the SW and RF lesions. (TIF) [file pone.0116017.s009.tif]
