# Supplementary material for: Development of a Novel Shock Wave Catheter Ablation System -The First Feasibility Study in Pigs-
Source: PLoS One. 2015 Jan 29;10(1):e0116017. doi: 10.1371/journal.pone.0116017 (PMC4310588; doi:10.1371/journal.pone.0116017)
Supplement: S1 Table — (DOCX) [file pone.0116017.s010.docx]

**Table S1. Number of Pigs and Application Sites in Each Study**

| Studies | Pigs (n) | Application sites (n) | | |
| --- | --- | --- | --- | --- |
|  |  | SW | RF | Sham |
| Epicardial ablation study |  |  |  |  |
| SW Pressure threshold study | 11 |  |  |  |
| 20-25 MPa |  | 16 | - | - |
| 30-35 MPa |  | 16 | - | - |
| 40-45 MPa |  | 16 | - | - |
| SW Duration study | 3 |  |  |  |
| 30 sec. |  | 16 | - | - |
| 60 sec. |  | 16 | - | - |
| 120 sec |  | 16 | - | - |
| Acute study | 14 | 96 | 16 | 16 |
| Time course study |  |  |  |  |
| Day 1 | 3 | 16 | 3 | - |
| Day 2 | 4 | 13 | 2 | - |
| Day 7 | 6 | 13 | 3 | - |
| Survival study | 13 | 42 | 8 |  |
| Total | 27 | 138 | 24 | 16 |
| Endocardial ventricular ablation study | 3 | 6 | 6 | - |
| Endocardial AV node ablation study |  |  |  |  |
| Acute study | 11 | 5 | 3 | 3 |
| Survival study | 15 | 9 | 3 | 3 |
| Total | 26 | 14 | 6 | 6 |
