# Supplementary material for: Development of a Novel Shock Wave Catheter Ablation System -The First Feasibility Study in Pigs-
Source: PLoS One. 2015 Jan 29;10(1):e0116017. doi: 10.1371/journal.pone.0116017 (PMC4310588; doi:10.1371/journal.pone.0116017)
Supplement: S2 Table — (DOCX) [file pone.0116017.s011.docx]

**Table S2. Electrophysiological Results in the Acute and Long-term Studies**

| Pigs | Heart rate (pulses/min) | | | Atrial-His interval (msec) | | | Wenckebach rate (pulses/min) | | | AV nodal function | | F/U(days) |
| --- | --- | --- | --- | --- | --- | --- | --- | --- | --- | --- | --- | --- |
|  | Pre | Post | Chronic | Pre | Post | Chronic | Pre | Post | Chronic | Post | Chronic |  |
| **Acute study** | | | | | | | | | | | | |
| Sham 1 | 113 | 118 | - | 81 | 81 | - | 230 | 240 | - | Normal | - | - |
| Sham 2 | 99 | 104 | - | 81 | 78 | - | 220 | 220 | - | Normal | - | - |
| Sham 3 | 83 | 118 | - | 64 | 65 | - | 260 | 270 | - | Normal | - | - |
| RF 1 | 91 | < 30 | - | 88 | AHB | - | 220 | CAVB | - | CAVB | - | - |
| RF 2 | 109 | < 30 | - | 76 | AHB | - | 240 | CAVB | - | CAVB | - | - |
| RF 3 | 108 | < 30 | - | 86 | AHB | - | 230 | CAVB | - | CAVB | - | - |
| SW 1 | 103 | 179 | - | 84 | JT | - | 260 | 280 | - | JT | - | - |
| SW 2 | 107 | 87 | - | 98 | AHB | - | 240 | CAVB | - | CAVB | - | - |
| SW 3 | 97 | 52 | - | 81 | AHB | - | 260 | CAVB | - | CAVB | - | - |
| SW 4 | 96 | 145 | - | 75 | JT | - | 230 | 200 | - | JT | - | - |
| SW 5 | 121 | 85 | - | 84 | AHB | - | 260 | CAVB | - | CAVB | - | - |
| Pigs | Heart rate (pulses/min) | | | Atrial-His interval (msec) | | | Wenckebach rate (pulses/min) | | | AV nodal function | | F/U(days) |
|  | Pre | Post | Chronic | Pre | Post | Chronic | Pre | Post | Chronic | Post | Chronic |  |
| **Long-term study** | | | | | | | | | | | | |
| Sham 4 | 142 | 129 | 104 | 88 | 87 | 85 | 250 | 250 | 230 | Normal | Normal | 11 |
| Sham 5 | 83 | 93 | 93 | 85 | 85 | 84 | 260 | 260 | 240 | Normal | Normal | 14 |
| Sham 6 | 100 | 103 | 83 | 71 | 72 | 73 | 230 | 230 | 250 | Normal | Normal | 10 |
| RF 4 | 82 | 39 | 51 | 87 | AHB | AHB | 190 | CAVB | CAVB | CAVB | CAVB | 10 |
| RF 5 | 119 | < 30 | 61 | 86 | AHB | AHB | 210 | CAVB | CAVB | CAVB | CAVB | 11 |
| RF 6 | 120 | < 30 | < 30 | 85 | AHB | AHB | > 300 | CAVB | CAVB | CAVB | CAVB | 14 |
| SW 6 | 110 | 62 | 88 | 70 | AHB | 134 | 230 | < 130 | 110 | 2:1 AVB | 1°AVB | 14 |
| SW 7 | 105 | < 30 | 72 | 82 | AHB | AHB | 230 | CAVB | CAVB | CAVB | CAVB | 15 |
| SW 8 | 106 | 71 | 79 | 77 | AHB | AHB | 230 | CAVB | CAVB | CAVB | CAVB | 7 |
| SW 9 | 97 | 73 | 71 | 80 | AHB | AHB | 250 | < 140 | Ad-AVB | 2:1 AVB | Ad-AVB | 8 |
| SW 10 | 138 | 96 | 70 | 75 | AHB | AHB | 240 | CAVB | CAVB | CAVB | CAVB | 8 |
| SW11 | 104 | 63 | 53 | 82 | AHB | AHB | 240 | CAVB | CAVB | CAVB | CAVB | 14 |
| Pigs | Heart rate (pulses/min) | | | Atrial-His interval (msec) | | | Wenckebach rate (pulses/min) | | | AV nodal function | | F/U(days) |
|  | Pre | Post | Chronic | Pre | Post | Chronic | Pre | Post | Chronic | Post | Chronic |  |
| **Long-term study** | | | | | | | | | | | | |
| SW12 | 82 | 116 | 70 | 97 | AHB | AHB | 230 | CAVB | CAVB | CAVB | CAVB | 15 |
| SW13 | 100 | 55 | 105 | 94 | AHB | 156 | 270 | < 110 | 160 | 2:1 AVB | 1°AVB | 14 |
| SW14 | 105 | 89 | 70 | 97 | AHB | AHB | 240 | CAVB | CAVB | CAVB | CAVB | 18 |

Pre: Measurements before procedure

Post: Measurements 15 min after last application

Chronic: Measurements before euthanasia after each follow up period

JT: Junctional tachycardia

AHB: Atrial-His block

AVB: Atrioventricular block

Ad-AVB: Advanced AV block

CAVB: Complete AV block

F/U: Follow up period.
